# Insulin-associated Weight Gain in Type 2 Diabetes and Its Relation with Caloric Intake

**DOI:** 10.7759/cureus.5275

**Published:** 2019-07-30

**Authors:** Nadeem Naeem, Abdul Basit, Ambreen Shiraz, Awn Bin Zafar, Nida Mustafa, Shaista Ali Siddique, Asher Fawwad

**Affiliations:** 1 Medicine, Baqai Institute of Diabetology and Endocrinology, Baqai Medical University, Karachi, PAK; 2 Biochemistry, Baqai Medical University, Karachi, PAK; 3 Research, Baqai Institute of Diabetology and Endocrinology, Baqai Medical University, Karachi, PAK; 4 Miscellaneous, Baqai Institute of Diabetology and Endocrinology, Baqai Medical University, Karachi, PAK

**Keywords:** weight gain, insulin, type 2 diabetes

## Abstract

Objective

The aim of this study was to observe the weight change in a patient with type 2 diabetes initiated on insulin therapy and the relation of weight gain with caloric intake.

Methods

This retrospective longitudinal follow-up study was conducted at the Baqai Institute of Diabetology and Endocrinology (BIDE), a tertiary care hospital of Karachi, Pakistan. Records of 917 patients attending the tertiary care diabetic clinic were retrieved from January 2009 to May 2016 from the Hospital Management System (HMS). Subjects were divided into two groups: group A consisted of subjects on oral hypoglycemic agents (OHA), while group B consisted of subjects on insulin therapy with OHA. Change in weight, change in HbA1c, and change in calories intake were calculated by examining data at baseline and end-line visit of the study.

Results

Group B showed significantly higher weight gain than group A (48.3% vs 24.8%). Insulin therapy with OHA (OR (95% CI = 1.78(1.05-3.02)), increased caloric intake (OR [95% CI = 1.98(1.093.60)]) and decreased HbA1c (OR [95% CI = 0.44(0.24-0.79)]) were the only factors identified as significant predictors of weight gain.

Conclusion

It is concluded that type 2 diabetic subjects, especially on insulin treatment, gain weight due to increase or unadvised caloric intake. Long-term multicenter studies are needed to ascertain the findings of this study.

## Introduction

Diabetes mellitus is a chronic metabolic disease characterized by either insulin deficiency or resistance of body to its action or both. The International Diabetes Federation (IDF) Atlas 2017 reports a prevalence of 8.8% around the globe [1]. Four out of five diabetics live in middle- or low-income countries with the Middle East and North Africa (MENA) region leading the prevalence table with around 11 percent [1]. The global prevalence has nearly doubled since 1980, showing a fast doubling rate as compared to other non-communicable diseases worldwide [2]. Diabetes mellitus took five million lives in a single calendar year in 2017, which is more than tuberculosis, malaria, and HIV combined [1]. Obesity with the diabetes is a co-morbid causing risk of complications to rise many folds. It is important to consider the weight effects of antidiabetic agents prior to initiation, as different antidiabetic agents impact weight differently [3]. Weight gain during insulin therapy can be a challenging problem in already overweight patients with type 2 diabetes mellitus affecting treatment compliance and long-term prognosis [4]. More importantly, it is also proven that weight reduction improves long-term glycemic control, making weight gain a very annoying side effect of antidiabetics both for endocrinologists treating it and patients [5-6]. Zoppini et al found an association between variability in body weight and glycemic control over a 10-year follow-up period and saw increased mortality in older patients with type 2 diabetes with worsening of these parameters [7]. Weight change in people with diabetes may be unintentional and is related to not only antidiabetic agents but also to uncontrolled sugars, sedentary lifestyle, and caloric intake, making these factors important variables affecting the main outcome variable [8-9]. Hence, clinicians should keep it in mind when working on antidiabetics-induced weight gain.

Pharmacological agents used in the treatment of diabetes directly contribute to weight gain through their glucose-lowering mechanisms [10-11]. Treatment with certain classes of therapies is associated with more weight gain than other type of therapies crowning insulin on top of the list. Sulfonylureas are insulin secretagogues that lead to minimal weight gain, compared with insulin, though they lower glycemia through many of the same mechanisms that occur with insulin use [12]. The mechanisms responsible for insulin-induced weight gain are varied, complex, and unclear [13]. A comparative study showed that subcutaneous delivery of insulin leads to more weight gain than intraperitoneal delivery [14]. Preclinical research has shown that insulin has a role in the central nervous system, where it regulates satiety signals and suppresses appetite, and it is suggested that these functions may be impaired in type 2 diabetes [15]. It seems to be a very important factor in weight gain when insulin is given therapeutically in disturbed satiety signal type 2 Diabetics. It is also an important fact to consider that not all the patients on insulin gain their weight, even some lose weight too, which is an interesting finding and need to be sorted out by finding the exact cause of weight gain in patients on insulin [16]. Another interesting finding is that most of the hypoglycemic agents well known for their weightloss potential have got one common side effect, which can be the most important factor for their potential of weight loss and that is either their gastrointestinal side effects or negative impact on glucose absorption which then ultimately causes a decrease in the dietary intake [17-18].

In this study, we tested the hypothesis that initiation of insulin therapy would be associated with an increase in weight gain as compared to oral hypoglycemic agents secondary to increased appetite and caloric intake.

## Materials and methods

This retrospective longitudinal follow-up study was conducted at the Baqai Institute of Diabetology and Endocrinology (BIDE), a tertiary care hospital of Baqai Medical University, Karachi, Pakistan from the duration of January 2009 to May 2016. Ethical approval for the study was obtained from the institutional review board (IRB) of BIDE.

A total of 917 cases were included in this study on the basis of inclusion criteria, that is, 1) age of 18 years and above, 2) being diagnosed with type 2 diabetes, 3) at least two visits during follow-up and the first and last visit included visit to dietician, and 4) having complete medical record in health management system (HMS) software of the institute. Subjects with other than type 2 diabetes and incomplete data records were excluded from the study. The records of subjects under study included demographical, anthropometrical, clinical, biochemical and dietary parameters. All subjects were given standard activity advice by the physician during follow-up.

The selected subjects were divided into two groups. Group A consisted of subjects on oral hypoglycemic agents (OHA), while group B consisted of subjects on insulin therapy along with OHA. Subjects who were on insulin therapy were mostly treated by free mixed insulin followed by basal human, premixed insulin, and analogs.

Caloric intake was calculated by American dietician food exchange list. Body mass index (BMI) was calculated as weight in kilograms divided by height in metre squared (kg/m^2^). Change in weight, change in HbA1c, and change in calories intake were calculated by examining data at baseline and end-line visit of the study. The change in weight was categorized into three groups as decrease or no weight change (BMI difference equal to or less than 0), slight weight gain (BMI difference ≥0.1 to ≤1) and substantial weight gain (BMI difference ≥1.01). Change in calories was categorized as same or decreased caloric intake (if the difference was zero or negative) and increased caloric intake (if the difference was positive). Likewise, the change in HbA1c was categorized as decreased or no change in HbA1c (if the difference was zero or negative) and increased HbA1c level (if the difference was positive).

Statistical analysis

The Statistical Package for Social Sciences (SPSS) 20 software program was used for data analysis. The chi-square test was applied to compare differences among the groups. Logistic regression was used to investigate the associations of the binary dependent variable “weight change” with continuous or categorical independent variables. Using backward selection, variables that remained significant were retained in the final model. *P*-value <0.05 was considered statistically significant.

## Results

Out of 917 type 2 diabetic subjects, 463 were males and 454 were females. Characteristics of subjects at the baseline visit are shown in Table 1. The mean age of subjects was 49.69 ± 10.63 years, Duration of diabetes was 12.93±7.16 years. Group A consisted of 11.9% subjects, while group B consisted of 88.12% subjects.

**Table 1:** Characteristics of subjects at baseline visit

Parameters	Mean ± SD or n (%)
N	917
Age (years)	49.69±10.63
Gender	
Male	463(50.5%)
Female	454(49.5%)
BMI (kg/m^2^)	28.88±5.13
Marital status	
Married	868(95.7%)
Single	39(4.3%)
Smoking habit	
No	820(90%)
Yes	97(10%)
Systolic BP (mmHg)	130.14±19.45
Diastolic BP (mmHg)	81.36±10.31
Family history of DM	
No	213(28.7%)
Yes	704(71.3%)
Duration of DM (years)	12.93±7.16
Duration of follow-up (years)	3.20±1.71
Treatment group	
Oral	109(11.9%)
Oral+ Insulin	808(88.1%)
HbA1c (%)	9.63±2.17
Creatinine (mg/dl)	1±0.27
Cholesterol (mg/dl)	180.09±43.97
LDL (mg/dl)	107.02±33.83
HDL (mg/dl)	37.94±9.67
Triglyceride (mg/dl)	193.89±147.58

Group B showed significantly higher weight gain than group A (48.3% vs 24.8%). It was also observed that in group B, 59.7% subjects had increased caloric intake whereas in group A, 54.1% subjects had increase caloric intake. This difference was not statistically significant (Table 2).

**Table 2:** Association of treatment type with weight change and change in caloric intake Data presented as *n *(%); *p*-value < 0.05 was considered statistically significant.

Parameters		Group A Oral therapy (OHA)	Group B Insulin therapy with OHA	P-value	Overall
Weight change	Decreased or no weight change	60(55%)	260(32.2%)	<0.0001	320(34.9%)
	Slight weight gain	22(20.2%)	158(19.6%)		180(19.6%)
	Substantial Weight gain	27(24.8%)	390(48.3%)		417(45.5%)
Change in caloric intake	Same or decreased	50(45.9%)	326(40.3%)	0.271	376(41%)
	Increased	59(54.1%)	482(59.7%)		541(59%)

In Table 3, treatment groups were stratified as change in caloric intake and change in the HbA1c level. It was found that in group A 30.5% subjects increased their caloric intake and gained weight while 18% gained their weight with same or decreased caloric intake. Similarly, in group B, 49.8% subjects had increased caloric intake and gained weight while 46% subjects had same caloric intake but still gained weight. No significant difference was observed in terms of weight change with change in caloric intake in both groups.

**Table 3:** Association between weight change, change in calories intake, and change in HbA1c level in treatment types Data presented as *n* (%); *p*-value < 0.05 was considered statistically significant.

Treatment group	Change in caloric intake	Decreased or no weight change	Slight weight gain	Weight gain	P-value
Group A	Same or decreased	33(66%)	8(16%)	9(18%)	0.104
	Increased	27(45.8%)	14(23.7%)	18(30.5%)	
Group B	Same or decreased	112(34.4%)	64(19.6%)	150(46%)	0.501
	Increased	148(30.7%)	94(19.5%)	240(49.8%)	
Overall	Same or decreased	145(38.6%)	72(19.1%)	159(42.3%)	0.138
	Increased	175(32.3%)	108(20%)	258(47.7%)	
	Change in HbA1c				
Group A	Decreased/no change	6(40%)	0(0%)	9(60%)	0.576
	Increased	8(50%)	4(25%)	4(25%)	
Group B	Decreased/no change	26(26%)	23(23%)	51(51%)	0.021
	Increased	41(41.41%)	20(20.2%)	38(38.38%)	
Overall	Decreased/no change	32(27.83%)	23(20%)	60(52.17%)	0.019
	Increased	49(42.61%)	24(20.87%)	42(36.52%)	

When comparing weight change with change in the HbA1c level in the treatment groups, it was noted that, in group A, 60% of subjects maintained their HbA1c level and gained weight over the follow-up period, whereas 25% subjects got weight gain with elevated HbA1c level (*P* > 0.05).

However, in group B, the majority of subjects who maintained their HbA1c level had significantly gained their weight (*P*-value < 0.05).

In Table 4, univariate analysis showed that weight gain was associated with oral and insulin treatment, decreased HbA1c, and decreased diastolic blood pressure (*P*-value < 0.05). Multivariate analysis demonstrated that caloric intake, use of insulin with oral hypoglycemic, and decreased HbA1c level remained associated with weight gain.

**Table 4:** Logistic regression demonstrating the association of weight change with other variables *Variables with *P*-value<0.25 were included in the multivariate regression model.

Variables		Univariate regression		Multivariate regression	
		OR (95% CI)	P-value	OR (95% C.I)	P-value
Treatment	Oral therapy	1	<0.0001	1	0.031
	Oral and insulin therapy	2.9(1.72-3.87)		1.78(1.05-3.02)	
Gender	Female	1	0.722	-	-
	Male	1.05(0.80-1.37)			
Change in calories	Decreased/ no change	1	0.052	1	0.023
	Increased	1.31(0.99-1.72)		1.98(1.09-3.60)	
Change in HbA1c	Decreased/ no change	1	0.02	1	0.006
	Increased	0.51(0.29-0.90)		0.44(0.24-0.79)	
Marital status	Married	1	0.227	-	-
	Single	1.57(0.75-3.26)			
Smoking habit	No	1	0.1	-	-
	Yes	0.63(0.40-1.08)			
Alcoholic	No	1	0.106	-	-
	Yes	0.24(0.04-1.34)			
Family history of DM	No	1	0.287	-	-
	Yes	1.19(0.85-1.67)			
Age		0.99(0.97-1.00)	0.186	-	-
Duration of DM		1.01(0.99-1.03)	0.293	-	-
Insulin duration		0.98(0.93-1.02)	0.4	-	-
Systolic BP		0.99(0.98-1.00)	0.109	-	-
Diastolic BP		0.98(0.97-1.00)	0.044	-	-

## Discussion

Insulin therapy initiation has long been delayed in most of the type 2 diabetics because of its weight-gaining potential, especially for those who are already obese. It gives an upper edge to the oral hypoglycemic in obese diabetics because these drugs are called weight neutral most of the times. The results of this study opened a new gate to researchers and fact seekers regarding this long-standing relationship between weight gain and insulin.

In this study, the finding of 48.3% subjects on insulin falling in the category of significant weight gain is in agreement with other previous studies, but the new variable of increased caloric intake in significant weight gain individuals has never been tested before in such a large sample size [12-13]. Another finding of this study is significant weight gain in 24.8% of the participants who were on oral hypoglycemic which is not in agreement with most of the studies till date stating the most of the oral hypoglycemic as weight neutral or slight weight gain potential drugs [12,19-21].

In most of the patients of both groups with significant weight gain, one similar finding was a significant increase in caloric intake which is not a common variable tested in most of the earlier studies with sufficient sample size.

This finding greatly emphasizes the importance of individualized diet plans with caloric counting and diabetic education in patients on insulin specially and oral hypoglycemic generally. Structured intensive diabetes education programs (SIDEPs) can motivate and empower patients to take control of their disease and have been associated with improved glycemic control, better weight management, and better adherence on insulin. Ko et al. proved the point in their study on type 2 diabetics who underwent an in-patient SIDEP versus hospitalized patients aiming for glycemic control without intensive education, the group receiving intensive education had significantly improved HbA1c levels, less frequent subsequent hospitalizations, and improved adherence to self-care behavior, the most challenging part of this education programme is deciding a food plan acceptable to both [22]. Nutrition therapy has an integral role in overall diabetes management, and each person with diabetes should be actively engaged in education, self-management, and treatment planning with his or her health care team, including the collaborative development of an individualized eating plan [23-24]. All individuals with diabetes should receive individualized medical nutrition therapy (MNT), preferably provided by a registered dietitian who is knowledgeable and skilled in providing diabetes-specific MNT. MNT delivered by a registered dietitian is associated with HbA1c decrease of 0.3-1% for people with type 1 diabetes and 0.5% to 2% for people with type 2 diabetes [25-27].

Worsening of glycemic control in patients with stable or decreased weight as evident by worsened HbA1c levels in those groups is not a finding which is in consensus with most of the earlier studies stating that weight loss causes improved glycemic control, but this finding may be related to suboptimal insulin or oral regimen causing suboptimal glycemic control with less weight gain or even weight loss, which is a proven fact by some studies [28-29].

Multivariate analysis showing that the increase in caloric intake and decreased HbA1c level remains associated with weight gain in addition to the use of insulin, which is again proving our point in the study. For people with diabetes on insulin therapy, education on how to use carbohydrate counting and in some cases fat and protein gram estimation to determine mealtime insulin dosing can improve glycemic control and prevent excessive weight gain. A simple and effective approach to hypoglycemia and weight management emphasizing portion control and healthy food choices may be helpful if standard MNT approach is not possible [30].

Limitations and strength

This study is of great significance because it is one of the very few studies known to discuss the role of caloric intake in a well-known side effect of insulin therapy. And it can help in controlling the weight of the patients on insulin therapy thus preventing the initiation of a vicious cycle of weight gain and then uncontrolled diabetes.

This study has a few limitations. First, this was a retrospective study, and the influence of unmeasured confounders on the results such as family history of obesity and physical activity of the patients is unknown.

Unfortunately, our groups did not include patients on the newer treatments in sufficient numbers suitable for analysis, for example, glucagon-like peptide-1 receptor agonists and SGLT2 blockers. We also did not sub-divide the groups on the basis of the type of insulin and the type of oral agents used.

Another limitation of this study is the difference in the number of subjects in groups A and B. As the follow-up of patients on oral hypoglycemic agents is usually long, hence we could not find a large number of patients on oral agents with at least two visits to dietician in a time span.

## Conclusions

It is concluded that type 2 diabetic subjects, especially on insulin treatment, gain weight due to unadvised and increased caloric intake. This finding emphasizes the importance of individualized diet plans with caloric counting and diabetic education in patients on insulin specially and oral hypoglycemic generally. This study further requires long-term follow-up with the dietician and its implementation in various other hospitals.
